# Commentary on “Co-Occurrence of Pituitary Adenoma With Suprasellar and Olfactory Groove Meningiomas”

**DOI:** 10.29252/NIRP.BCN.8.6.513

**Published:** 2017

**Authors:** Lorenzo Curtò, Salvatore Cannavò

**Affiliations:** 1 Peloritan Academy of Periculants, University of Messina, Messina, Italy.; 2 Department of Clinical and Experimental Medicine, Unit of Endocrinology, University of Messina, Messina, Italy.

**Keywords:** Pituitary adenoma, Suprasellar meningioma, Olfactory groove meningioma, Magnetic resonance imaging, Growth hormone, Somatostatin analog

## Abstract

Recently, *Basic and Clinical Neuroscience* published an article by Lim et al. (2016) entitled *Co-occurence of Pituitary Adenoma with Suprasellar and Olfactory Groove Meningiomas*. They claimed it as the first case of co-occurence of these two malignancies. However, to our knowledge, this is not the first case reported in this regard. We reported the same case scenario in a 61-year-old woman referred to our outpatient clinic in 2007. In this commentary, we are going to discuss our reported case and present a brief review over co-occurence of intracranial meningioma with pituitary adenoma.

We read with interest the case report by Lim et al. (2016) entitled “Co-occurrence of Pituitary Adenoma with Suprasellar and Olfactory Groove Meningiomas” recently published in Basic and Clinical Neuroscience. The authors described the first reported case of synchronous non-functioning pituitary macroadenoma with suprasellar and olfactory groove meningiomas in a 65-year-old woman not previously irradiated.

However, in a paper published in Pituitary, we reported the first case ever diagnosed of simultaneous occurrence of GH-secreting pituitary adenoma, intracranial meningioma and two intracavernous asymptomatic aneurysms in a 61-year-old woman who was referred to our outpatient clinic reporting severe obesity and appearance of typical acromegalic features 13 years after a right frontal craniotomy for a pituitary tumor (Curtò et al., 2007). Endocrine evaluation showed increased serum GH and IGF-1 levels without GH suppression during standard OGTT while serum gonadotropins levels were very low, despite menopausal age.

In the case by Lim et al., (2016) endocrine evaluation suggested the presence of a non-functioning pituitary adenoma. Preoperative contrast-enhanced MRI revealed a large pituitary lesion (16×13×20 mm) consistent with pituitary macroadenoma and, immediately adjacent to it, a homogenous enhancing lesion (15×13×13 mm) arising from tuberculum sellae and compressing the optic apparatus. A third small homogenously enhancing lesion (6×5 mm) with a dural tail was found along the olfactory groove, consistent with an olfactory groove meningioma. The patient underwent extended endoscopic transsphenoidal neurosurgery with resection both of mass pathologically consistent with a pituitary adenoma and the suprasellar meningioma while the asymptomatic olfactory groove meningioma was conservatively managed with serial MRI evaluation.

In our case, not cited in the case report by Lim, MRI showed a homogeneously enhancing intrasellar pituitary macroadenoma (12×10 mm) extending superiorly with visual pathways compression and bilateral intracavernous invasion, and simultaneous coexistence of a large intracavernous aneurysm of the right Internal Carotid Artery (ICA) adjacent to the pituitary adenoma. The presence of the right ICA aneurysm was confirmed by MRI angiography with 3D evaluation by Maximum Intensity Projection (MIP) reconstruction. Somatostatin Analog (SSA) treatment normalized GH and IGF-1 levels in a few weeks. Eight months later, the patient underwent a balloon ICA occlusion by endovascular approach with disappearance of the right ICA aneurysm. After one year, a new MRI confirmed the presence of the pituitary adenoma along with the appearance of a right frontal meningioma and a new ICA aneurysm on the left side. The patient preferred an expectation management refusing the surgical option. The last MRI confirmed the presence of three distinct lesions, showing the growth both of meningioma and of the left ICA aneurysm.

Coexistence of pituitary lesion and other brain diseases has been frequently reported in literature. Moreover, an elevated co-prevalence of independent primary tumors has been found in patients with benign and malignant pituitary tumors and in their relatives (Couldwell, & Cannon-Albright, 2014). Association of pituitary adenoma with intracranial tumor is not a rare event, usually regarding patients previously irradiated for a pituitary mass. Meningiomas are one of the most frequent primary intracranial tumors, accounting for 15%–25% of all CNS neoplasms, but the simultaneous occurrence of pituitary adenoma and intracranial meningioma is, on the contrary, a quite rare event (Karsy, Sonnen, & Couldwell, 2015), usually reported not only in patients with functioning and non-functioning pituitary adenoma after radiotherapy (Partington, & Davis, 1990), but also in patients not previously irradiated, suggesting that this association may be a casual discovery without relationship between the two diseases. A possible role of GH or other growth factors in the appearance or growth of meningioma has also been hypothesized but this statetment is not fully supported. In our case, the appearance and growth of meningioma was observed despite effective octreotide treatment, suggesting that SSAs can play a growth-promoting role (De Menis et al., 2003).

In conclusion, in literature the association of intracranial meningioma with pituitary adenoma is a rarely reported surgical entity, but the simultaneous occurrence of multiple meningiomas and pituitary adenoma is an even more rare clinical event. Our case, described in 2007, represents the first case of an equally rare simultaneous occurrence of pituitary adenoma, intracranial meningioma and two intracavernous aneurysms adjacent to the pituitary adenoma and the first in which MRI allowed preoperative identification. In the presence of concomitant brain morbidities (benign and malignant lesions, and vascular diseases) treatment must be tailored to each patient. For this reason, the adequate knowledge of these coexistences, based on their location, is an essential precondition to plan a correct surgical approach, and to avoid life-threatening complications.
